# *Uwhangchungsimwon* Inhibits Oxygen Glucose Deprivation/Re-Oxygenation-Induced Cell Death through Neuronal VEGF and IGF-1 Receptor Signaling and Synaptic Remodeling in Cortical Neurons

**DOI:** 10.3390/antiox11071388

**Published:** 2022-07-18

**Authors:** Jin Young Hong, Hyunseong Kim, Changhwan Yeo, Wan-Jin Jeon, Junseon Lee, Seung Ho Baek, Yoon Jae Lee, In-Hyuk Ha

**Affiliations:** 1 Jaseng Spine and Joint Research Institute, Jaseng Medical Foundation, Seoul 135-896, Korea; vrt23@jaseng.org (J.Y.H.); biology@jaseng.org (H.K.); duelf2@jaseng.org (C.Y.); cool2305@jaseng.org (W.-J.J.); excikind@jaseng.org (J.L.); goodsmile@jaseng.org (Y.J.L.); 2 College of Korean Medicine, Dongguk University, 32 Dongguk-ro, Ilsandong-gu, Goyang-si 10326, Korea; 201912223@dongguk.ac.kr

**Keywords:** *Uwhangchungsimwon*, oxygen glucose deprivation/re-oxygenation, vascular endothelial growth factor, insulin-like growth factor-1, angiogenesis, neuroprotection, antioxidants, stroke

## Abstract

*Uwhangchungsimwon* (UCW), a multi-component herbal product, has long been used to treat vascular diseases such as headache, dizziness, high blood pressure, and stroke. Though the prophylactic actions of UCW are well known, insufficient experimental evidence exists on its effectiveness against stroke. Here, we investigated the mechanism underlying the efficacy of UCW in oxygen glucose deprivation/re-oxygenation (OGD/R)-injury to the primary cortical neurons using an in vitro ischemia model. Neurons secrete vascular endothelial growth factor (VEGF), which acts as a neurotrophic factor in response to an ischemic injury. VEGF modulates neuroprotection and axonal outgrowth by activating the VEGF receptors and plays a critical role in vascular diseases. In this study, cortical neurons were pretreated with UCW (2, 10, and 50 µg/mL) for 48 h, incubated in oxygen-glucose-deprived conditions for 2 h, and further reoxygenated for 24 h. UCW effectively protected neurons from OGD/R-induced degeneration and cell death. Moreover, the role of UCW in sustaining protection against OGD/R injury is associated with activation of VEGF-VEGFR and insulin-like growth factor 1 receptor expression. Therefore, UCW is a potential herbal supplement for the prevention of hypoxic-ischemic neuronal injury as it may occur after stroke.

## 1. Introduction

Stroke is a serious neurological condition caused by the interruption of blood supply that presents potentially life-threatening complications of sudden difficulty in movement, speaking, and vision, and dizziness, or lack of balance and coordination [1,2]. Fifteen million people who have suffered from stroke worldwide are living with permanent disabilities, and stroke prevalence is estimated to increase with the aging of society [3]. However, there are no definitive clinical treatment methods available. The well-known treatment strategy for stroke is to promote angiogenesis, neurogenesis, and neuroplasticity, which contribute to neurorestorative processes [4,5].

The initiation of angiogenesis can be regulated by angiogenic growth factors, including vascular endothelial growth factor (VEGF), insulin-like growth factor-1 receptor (IGF-1R), basic fibroblast growth factor, and angiopoietins, which are released by endothelial cells and neurons [6,7,8]. Angiogenesis is closely linked to neurogenesis during axon growth and neuronal recovery. Most studies have reported that VEGF expression has a potent effect on neurons as a direct angiogenic or neurotrophic factor that promotes neuronal survival, growth, and axonal outgrowth [9]. Therefore, enhancing the expression of angiogenic factors has been suggested as a therapeutic measure for reducing serious complications and resolving the symptoms after stroke.

Traditionally, *Uwhangchungsimwon* (UCW) has been used as a multi-component herbal product in Korea and other East Asian countries to treat and prevent brain-related diseases such as headache, dizziness, syncope, stroke, or convulsions [10]. Various pharmacological properties have been attributed to UCW based on experimental analysis, including brain protection and neurogenesis. Furthermore, in vivo effects of UCW were assessed in a chronic stress murine model, a social isolation-induced depressive-like model, and epilepsy, indicating its protective role in brain diseases. Its effectiveness was observed in stress-induced oxidative damage and in regulating the hypothalamic-pituitary-adrenal axis, stress-related hormones, serotonergic system, and brain-derived neurotrophic factor in mice [10,11,12].

Based on the previous studies that state UCW can be introduced as an herbal-based natural agent for the prevention and management of brain-related diseases, we hypothesized that UCW may have a potential preventive role against oxygen glucose deprivation/re-oxygenation-induced neuronal injury through VEGFR and IGF-1R activation. In particular, a previous study showed the transcriptional regulation of UCW for the vascular cell adhesion molecule-1 (VCAM-1) in the human endothelial cell line [13], which supports the mechanism of this hypothesis. Other studies showed that VCAM-1 is an angiogenesis-related marker [14] and that VEGF can activate the signal transduction pathways that regulate VCAM-1 expression [15]. Additionally, enhanced neovascularization and neurogenesis could be the essential mechanisms by which insulin-like growth factor-1 (IGF-1)/IGF-1R improves functional recovery after stroke [16].

Toward this, we used a hypoxic in vitro model of ischemic stroke and applied various doses of UCW to the cortical neurons. After validating the optimal concentration, we examined the therapeutic efficacy in reducing neuronal death, as well as in improving the expression of VEGFR and IGF-1R. This is the first report demonstrating the in vitro preventive effect of UCW in a hypoxic in vitro model of ischemic stroke, thus, providing evidence for the use of herbal-based natural agents in preventive care of stroke.

## 2. Materials and Methods

### 2.1. Primary Culture of Rat Cortical Neurons

The study was approved by the Jaseng Animal Care and Use Committee (JSR-2020-03-004). Sprague-Dawley rat embryos (embryonic day 17, Dae Han Bio Link, Chungbuk, Korea) were used to culture primary cortical neurons, as previously described [17,18]. Briefly, pregnant rats were sacrificed; the embryos were isolated and immediately placed in a petri dish containing cold Hank’s balanced salt solution (HBSS; Gibco BRL, Grand Island, NY, USA). The brain was cut with No. 5 fine forceps under a light microscope. The brainstem and cerebellar tissue were discarded, and the brain was separated into two hemispheres. The meninges were peeled off, and the hippocampus was removed with No. 5 fine forceps (Video S1). The cortical tissues were digested using a neural tissue dissociation kit (Miltenyi, Bergisch Gladbach, Germany), according to the manufacturer’s protocol, and centrifuged at 2000 rpm for 3 min at room temperature. Cell pellets were triturated in 1 mL of cortical neuron culture medium containing neurobasal medium (Gibco BRL) supplemented with 1% penicillin/streptomycin (Gibco BRL), 1% Gluta-MAX (Gibco BRL), and 2% B27 (Gibco BRL), and then seeded into poly-D-lysine (Gibco BRL) and laminin-coated 12 mm glass coverslips (Paul Marienfeld GmbH and Co., Lauda-Königshofen, Germany) in 24-well plates at 2 × 10^4^ cells/450 µL for immunocytochemistry, a glass-bottom black 24-well μ-Plate (82426; ibidi, Martinsried, Germany) for live imaging, 6-well plates at 2 × 10^6^ cells/1.8 mL for fluorescence-activated cell sorting analysis, 60 mm^2^ dishes at 4 × 10^6^ cells/2.7 mL for real-time PCR, and 96-well plates at 2 × 10^4^ cells/90 µL for cell viability assessment.

### 2.2. UCW Preparation

UCW was produced from 21 medicinal herbs: *Dioscorea batatas* Decne. (0.150 g/1 g), *Glycyrrhiza glabra* L. (0.107 g), *Panax ginseng* C.A. Mey. (0.054 g), *Typha orientalis* C. Presl (0.054 g), *Massa Medicata Fermentata* (0.054 g), *Cornu Bubali* (0.043 g), *Glycine max* subsp. *soja* (Siebold and Zucc.) H. Ohashi (0.038 g), *Cinnamomum cassia* (Nees and T. Nees) J. Presl (0.038 g), *Equus asinus* L., *Paeonia lactiflora* Pall. (0.032 g), *Liriope platyphylla* F.T. Wang and Tang (0.032 g), *Scutellaria baicalensis* Georgi (0.032 g), *Angelica gigas* Nakai (0.032 g), *Saposhnikovia divaricata* (Turcz.) Schischk. (0.032 g), *Atractylis japonica* (Koidz. ex Kitam.) Kitag. (0.032 g), *Bupleurum falcatum* L. (0.027 g), *Platycodon grandiflorum* A. (0.027 g), *Zizyphus jujuba* Mill. (0.168 g), *Bostaurus Linne* var. *domesticus* Gmelin (0.004 g), *Aquilaria agallocha* Roxb. (0.004 g), *Dryobalanops aromatica* C.F. Gaertn. (0.003 g). It was dried for 24 h at 70 °C in a dryer. After drying, the medicinal material was finely pulverized using a grinder. A stock solution was prepared at 10 mg/mL by dissolving the powdered mixture in phosphate buffered saline (PBS, Gibco BRL). The solution was filter-sterilized using a 0.45 μm polytetrafluoroethylene syringe filter (Advantec Co., Tokyo, Japan).

### 2.3. UCW Pretreatment and Oxygen Glucose Deprivation/Re-Oxygenation (OGD/R)

**Scheme 1:**
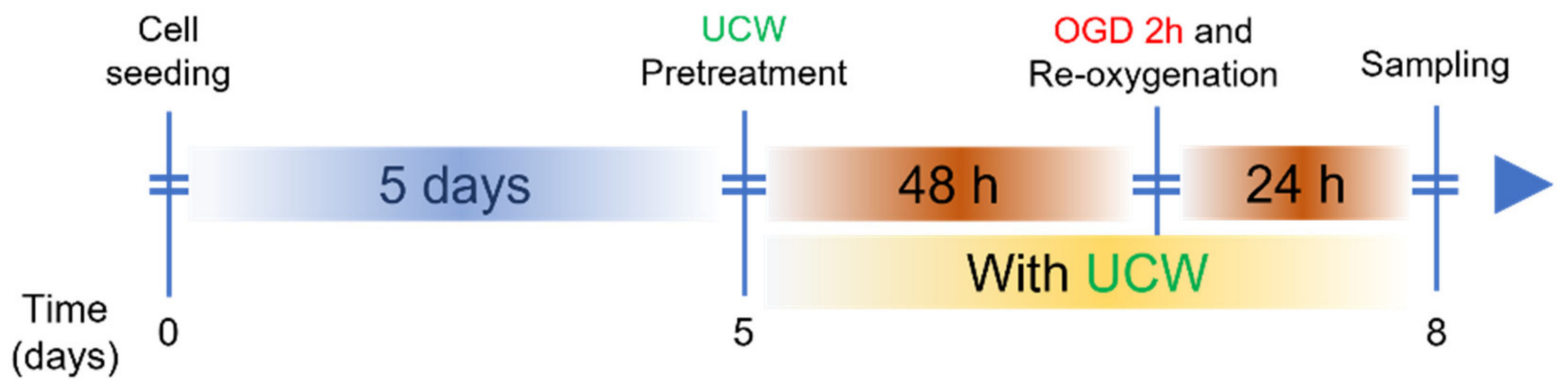
Flowchart showing experimental timeline of *Uwhangchungsimwon* (UCW) treatment and oxygen glucose deprivation/re-oxygenation (OGD/R).

The cells were maintained for 5 days in the cortical neuron culture medium (neurobasal medium, (Gibco BRL) with penicillin/streptomycin (1:100, Gibco BRL), B-27 (1:50, Gibco BRL), and GlutaMAX (1:100, Gibco BRL)). Media was changed every 2 days. The cells were then pretreated with UCW at concentrations of 2, 10, and 50 µg/mL for 48 h in the cortical neuron culture medium. After 48 h of treatment, the medium was replaced with glucose-free DMEM (Gibco BRL, Grand Island, NY, USA) containing UCW. Cells were then incubated in a hypoxia chamber containing 94% N_2_, 5% CO_2_, and 1% O_2_ for 2 h at 37 °C. Following the OGD with glucose-free DMEM for 2 h, the cells were exposed to a cortical neuron culture medium containing UCW at concentrations of 2, 10, and 50 µg/mL in a 5% CO_2_ incubator for 24 h. Samples were then divided into five groups: (1) Blank group; no-UCW pretreatment and OGD/R group; (2) OGD/R group; OGD/R only group; (3) UCW-2 group; 2 µg/mL UCW pretreated + OGD/R group; (4) UCW-10 group; 10 µg/mL UCW pretreated + OGD/R group; (5) UCW-50 group; 50 µg/mL UCW pretreated + OGD/R group. A timeline of the experimental design is described in Scheme 1.

### 2.4. Cell Counting Kit-8 (CCK-8) Assay

The CCK-8 assay (CCK-8; Dojindo, Kumamoto, Japan) was used to confirm the effect of UCW on cell viability following OGD/R-induced injury. First, cells were seeded onto 96-well plates at 2 × 10^4^ cells/90 µL and treated with various concentrations of UCW with or without OGD/R exposure. After incubation for 24 h, 10 µL of the CCK-8 solution was added to each well. After 4 h, absorbance was measured at 450 nm using a microplate reader (Epoch, BioTek, Winooski, VT, USA). Cell viability was calculated as the percentage of surviving neurons relative to the blank.

### 2.5. Live and Dead Assay

Neuronal viability was determined using a live/dead cell imaging kit (Thermo Fisher Scientific, Waltham, MA, USA) according to the manufacturer’s instructions. Briefly, the cells were incubated for 15 min at 37 °C in 100 µL of staining solution, which consisted of two dyes: calcein AM, staining the live cells (green), and BOBO-3 iodide (EthD-1), staining dead cells (red). After washing and mounting with a fluorescence mounting medium (Dako, Agilent, Santa Clara, CA, USA), ten random images per group were captured at 100× magnification using a confocal microscope (Eclipse C2 Plus, Minato, Tokyo, Nikon, Japan). The intensities of live and dead cells were measured using ImageJ software (1.37v, National Institutes of Health, Bethesda, MD, USA).

### 2.6. Live Imaging

Live cell-permeant Hoechst 33342 (0.5 µM/mL, Invitrogen, Grand Island, NY, USA) and live cell-impermeable propidium iodide (PI; 4 µM/mL, Invitrogen) were added to serum-free neurobasal medium. Live-cell imaging was performed every 30 min for 24 h at five sites per well using Tokai Hit, STX series (Controller: STXG, chamber: WSKMX, Shizuoka, Japan) with a confocal microscope under stage maintained at a temperature of 37 °C (top at 45.5 °C and bath at 41 °C) in 5% CO_2_.

### 2.7. Immunocytochemistry

The treated/untreated cells were fixed using 4% paraformaldehyde for 30 min and washed thrice with PBS. Cells were treated with 0.2% Triton X-100 in PBS for 5 min, washed, and blocked with 2% normal goat serum (NGS) in PBS for 1 h. The following primary antibodies were diluted in 2% NGS in PBS and incubated overnight at 4 °C; iNOS (1:100; Cell signaling, Beverly, MA, USA), MAP2 (1:1000; Synaptic Systems, Goettingen. Germany), VEGFR (1:500; Novus, Littleton, CO, USA), VEGF (1:400; Sigma Aldrich, St. Louis, MO, USA), IGF-1R (1:100; Invitrogen), GFAP (1:500; Sigma Aldrich), Synapsin1 (1:500; Invitrogen), PSD95 (1:100; Novus), Rhodamine Phalloidin (F-actin; 1:400; Invitrogen). After washing with PBS, secondary antibodies (FITC-conjugated goat anti-rabbit IgG, FITC-conjugated goat anti-mouse IgG, Rhodamine Red-X-conjugated goat anti-rabbit IgG, and Rhodamine Red-X-conjugated goat anti-mouse IgG, Alexa Fluor^®^ 594 AffiniPure Goat Anti-Guinea Pig IgG, Alexa Fluor^®^ 647-conjugated AffiniPure Goat Anti-Rabbit IgG, Alexa Fluor^®^ 647-conjugated AffiniPure Goat Anti-Mouse IgG; Jackson Immuno-Research Labs, West Grove, PA, USA) were diluted at 1:300 in 2% NGS and incubated for 2 h at room temperature (RT). After washing three times with PBS, the cells were mounted with a fluorescence mounting medium (Dako), and ten representative images were captured at 100× or 400× magnification with the same acquisition settings via confocal microscopy (Eclipse C2 Plus, Nikon, Tokyo, Japan). The average fluorescence intensity was quantitatively determined using the ImageJ software and compared with that of the OGD/R group.

### 2.8. Flow Cytometry

For reactive oxygen species (ROS) analysis, 2,7-dichlorofluorescin diacetate (DCFDA; Sigma Aldrich) was used to detect intracellular ROS levels. Briefly, we prepared a stock solution (5 mM by dissolving 9.3 mg DCFDA powder in high-quality 3.8 mL anhydrous dimethyl sulfoxide (DMSO, Sigma) and added 1 mL of 10 µM DCFDA solution to the cell pellet. The mean positive cell values, as determined by flow cytometry, were expressed as percentages relative to the OGD/R group.

### 2.9. Real-Time PCR

We evaluated changes in the mRNA expression levels of *iNOS*, *VEGFR*, *NF200*, *GAP43*, *IGF-1R*, and *VEGF* in each group using real-time PCR. Total RNA was isolated using the TRIzol reagent (Ambion, Thermo Fisher Scientific). Complementary DNA (cDNA) was synthesized from 1 µg of total RNA using oligo (dT) 20 primer and AccuPower RT PreMix (Bioneer, Daejeon, Korea). All primer pairs were designed using the UCSC Genome Bioinformatics and NCBI databases, and their sequences are listed in Table 1. Real-time PCR was performed using iQ SYBR Green Supermix (Bio-Rad, Hercules, CA, USA) on a CFX Connect Real-Time PCR Detection System (Bio-Rad). The cycling conditions were 40 cycles of initial denaturation at 95 °C for 3 min, denaturation at 95 °C for 15 s, and annealing at 60 °C for 30 s. All real-time PCR reactions were performed in triplicate or more. The expression of target genes was normalized to that of GAPDH and expressed as fold change relative to the OGD/R group.

### 2.10. Western Blotting

Cells were lysed in RIPA buffer (CellNest, Minato, Tokyo, Japan) containing protease inhibitor cocktail set III (1:1000, Millipore, Billerica, MA, USA). The concentration of the extracted protein was determined using a Pierce BCA Protein Assay Kit (Thermo Fisher Scientific) in accordance with the manufacturer’s protocol. The extracted proteins were separated using SDS-PAGE (20 μg proteins/lane), transferred to a polyvinylidene difluoride (PVDF) membrane, blocked with 5% DifcoTM skim milk (BD Biosciences, San Jose, CA, USA) in a buffer solution consisting of 1× tris-buffered saline (TBS, Bio-Rad) with 0.1% (*v*/*v*) Tween 20 (TBST), and probed with IGF-1R (1:1000, Cell signaling) and VEGFR (1:500; Novus). After overnight incubation at 4 °C, membranes were washed and then soaked in secondary solutions containing horseradish peroxidase-conjugated anti-rabbit or anti-mouse antibodies (1:2500, Abcam, Cambridge, UK). Detection was accomplished using ECL (Bio-Rad) and imaged using an Amersham Imager 600 (GE Healthcare Life Sciences, Piscataway, NJ, USA). Equivalent protein loading was verified by probing with β-actin.

### 2.11. Statistics

All results are expressed as mean ± standard error of the mean (SEM). Comparisons among groups were performed using one-way analysis of variance (ANOVA) with Tukey’s post-hoc analysis (GraphPad Prism, California, CA, USA). Differences were considered statistically significant if the *p*-value was # *p* < 0.05, ## *p* < 0.01, ### *p* < 0.001 or #### *p* < 0.0001 vs. the blank group and * *p* < 0.05, ** *p* < 0.01, *** *p* < 0.001, or **** *p* < 0.0001 vs. the OGD/R group.

## 3. Results

### 3.1. UCW Protects against the OGD/R-Induced Decrease in Cell Viability of Primary Cortical Neurons

**Figure 1:**
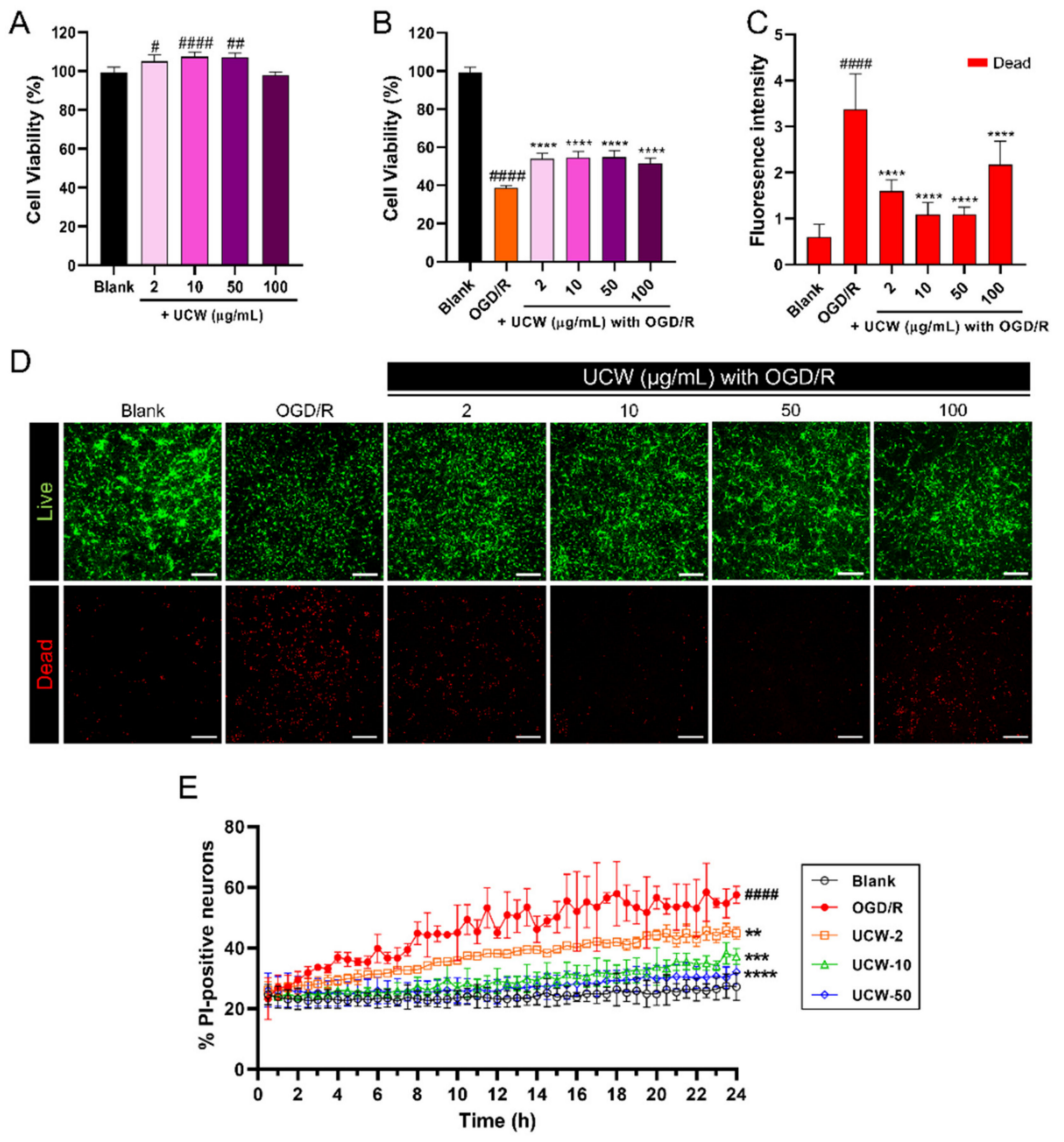
Protective effect of UCW against OGD/R-induced injury in primary cortical neurons. (**A**) Cell viability of primary cortical neurons pretreated with UCW for 48 h without OGD/R injury and (**B**) with OGD/R injury using CCK-8 assay (*n* = 10). (**C**) Fluorescence intensity of dead cells (red-stained) in neurons pretreated with UCW for 48 h with OGD/R injury (*n* = 10). (**D**) Representative images of live and dead assay for live (green) and dead (red) cells in blank, OGD/R injury, and UCW + OGD/R injury. White scale bar = 200 µm. (**E**) Live-cell imaging of propidium iodide (PI)-stained OGD/R-injured neurons pretreated with UCW at a concentration of 2, 10, and 50 µg/mL for 24 h (*n* = 6). Data are expressed as the mean ± SEM. Significant differences were analyzed via ordinary one-way analysis of variance (ANOVA) with Tukey’s post-hoc analysis as follows: ^#^ *p* < 0.05, ^##^ *p* < 0.01, and ^####^ *p* < 0.0001 vs. blank group; ** *p* < 0.01, *** *p* < 0.001, and **** *p* < 0.0001 vs. the OGD/R group.

To assess whether UCW treatment can prevent OGD/R-induced cell death in a concentration-dependent manner, we performed CCK-8 assays, live/dead assays, and live imaging in primary cortical neurons. After 5 days of culture, neurons were pretreated with UCW for 48 h prior to OGD/R exposure. It was observed that UCW was not toxic to neurons up to 50 µg/mL with a significantly increased viability. UCW in a concentration of 100 µg/mL showed decreasing trend, yet none were significant (Figure 1A). Furthermore, the effective doses of UCW were determined in neurons exposed to OGD/R. The CCK-8 assay showed that cell viability was significantly decreased after OGD/R exposure compared to that in the blank group; in contrast, UCW significantly increased cell viability up to 100 µg/mL (Figure 1B). We also investigated whether the CCK-8 results correlated with those for live/dead assays. In the UCW-only toxicity test, no significant changes were observed in the number of viable cells up to 100 µg/mL. Meanwhile, nonviable cells tended to increase at 100 µg/mL; however, the difference between the groups was not statistically significant (Figure S1). Exposure to OGD/R resulted in increased cell death which was significantly decreased up to 50 µg/mL in the UCW-pretreated OGD/R condition (Figure 1C,D). Cortical neurons without pretreatment showed an increase in PI staining post-OGD/R insult, which was normalized to Hoechst-stained cells. However, UCW pretreatment led to a reduction in PI-positive cells up to 50 µg/mL (Video S2). Quantitatively, apoptotic cell percentage, as assessed using PI staining and live imaging, was increased in cells after OGD/R injury, while UCW displayed a protective phenotype based on the reduction in apoptotic cells (Figure 1E).

### 3.2. UCW Attenuates OGD/R-Induced Oxidative Injury via Inhibition of iNOS-Mediated ROS Signaling in Primary Cortical Neurons

**Figure 2:**
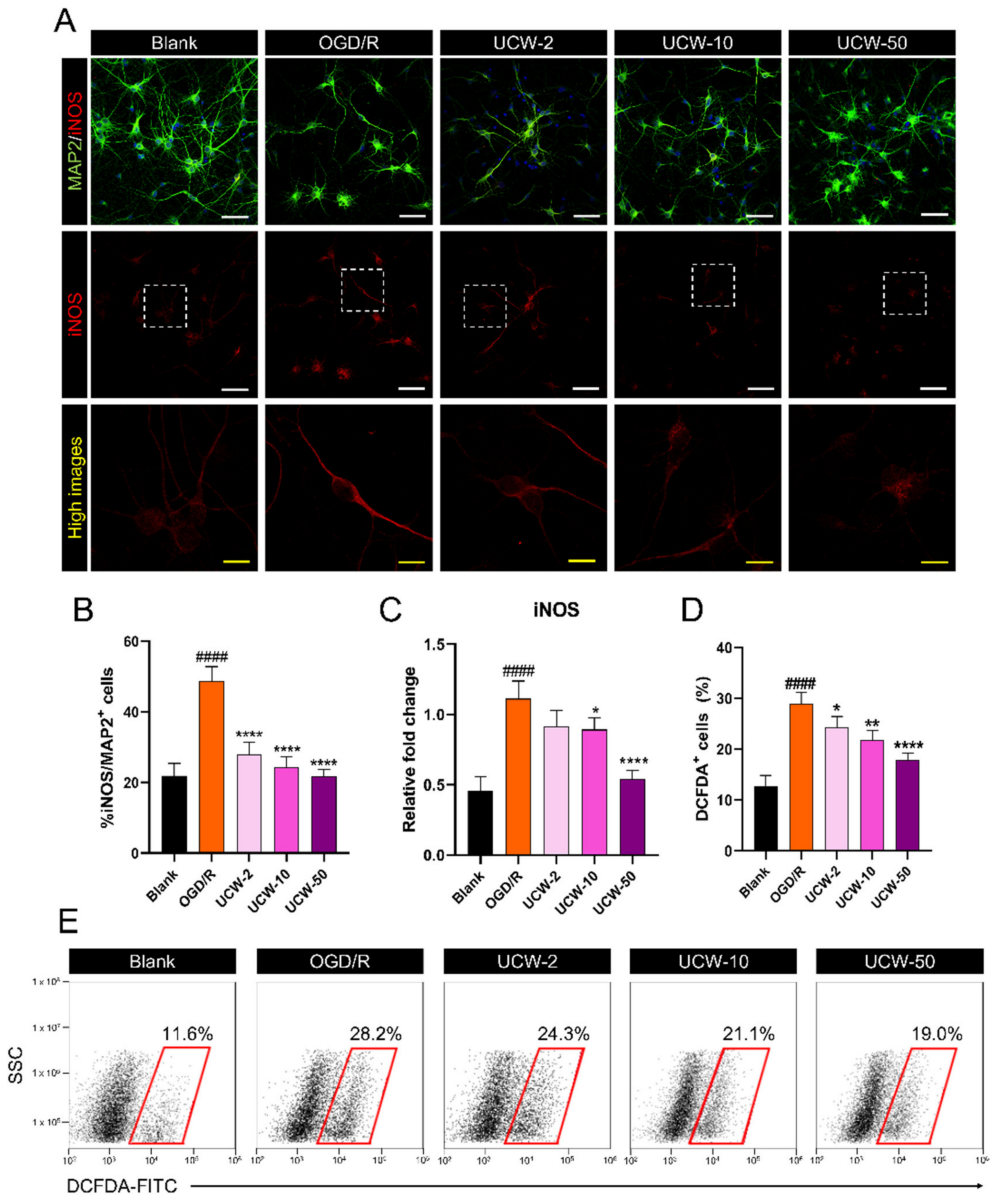
Antioxidant effect of UCW against OGD/R-induced oxidative injury via inhibition of iNOS-mediated ROS signaling. (**A**) Representative images of immunocytochemistry for MAP2 (green) and iNOS (red) in blank, OGD/R injury, UCW + OGD/R injury. White scale bar = 50 µm, yellow scale bar = 10 µm. (**B**) Quantitative analysis of iNOS+/MAP2+ cells estimated based on counts obtained from 10 non-overlapping fields in each group (*n* = 10). (**C**) The relative gene expression levels for *iNOS* mRNA using real-time PCR in each group (*n* = 4). (**D**) The percentage of H_2_DCFDA^+^ cells in each group by flow cytometry (*n* = 4). (**E**) Representative flow cytometry plot images of side scatter (SSC) versus H_2_DCFDA-FITC staining for intracellular ROS levels. Data are expressed as the mean ± SEM. Significant differences were analyzed via ordinary ANOVA with Tukey’s post-hoc analysis as follows: ^####^ *p* < 0.0001 vs. blank group; * *p* < 0.05, ** *p* < 0.01, and **** *p* < 0.0001 vs. OGD/R group.

OGD/R-induced oxidative stress is a major cause of neuronal death. We used immunocytochemistry to examine the effects of UCW on iNOS expression in OGD/R-injured neurons (Figure 2A). iNOS expression was significantly increased post OGD/R injury in MAP2-positive neurons, while UCW led to a significant decrease in iNOS in a dose-dependent manner (Figure 2B). This dose-dependent trend was also observed in the real-time PCR (Figure 2C). However, the *iNOS* mRNA level showed no significant difference between the OGD/R and UCW-2 groups, and this expression was significantly decreased in the UCW-10 and UCW-50 groups compared to the OGD/R group. Additionally, we evaluated the OGD/R-induced ROS generation by flow cytometry using H_2_DCFDA. The percentage of H_2_DCFDA-positive cells was significantly high when the neurons were exposed to OGD/R but was found to decrease following pretreatment with UCW in a dose-dependent manner (Figure 2D,E).

### 3.3. UCW Activates VEGF-VEGFR Signaling in OGD/R-Injured Primary Cortical Neurons

**Figure 3:**
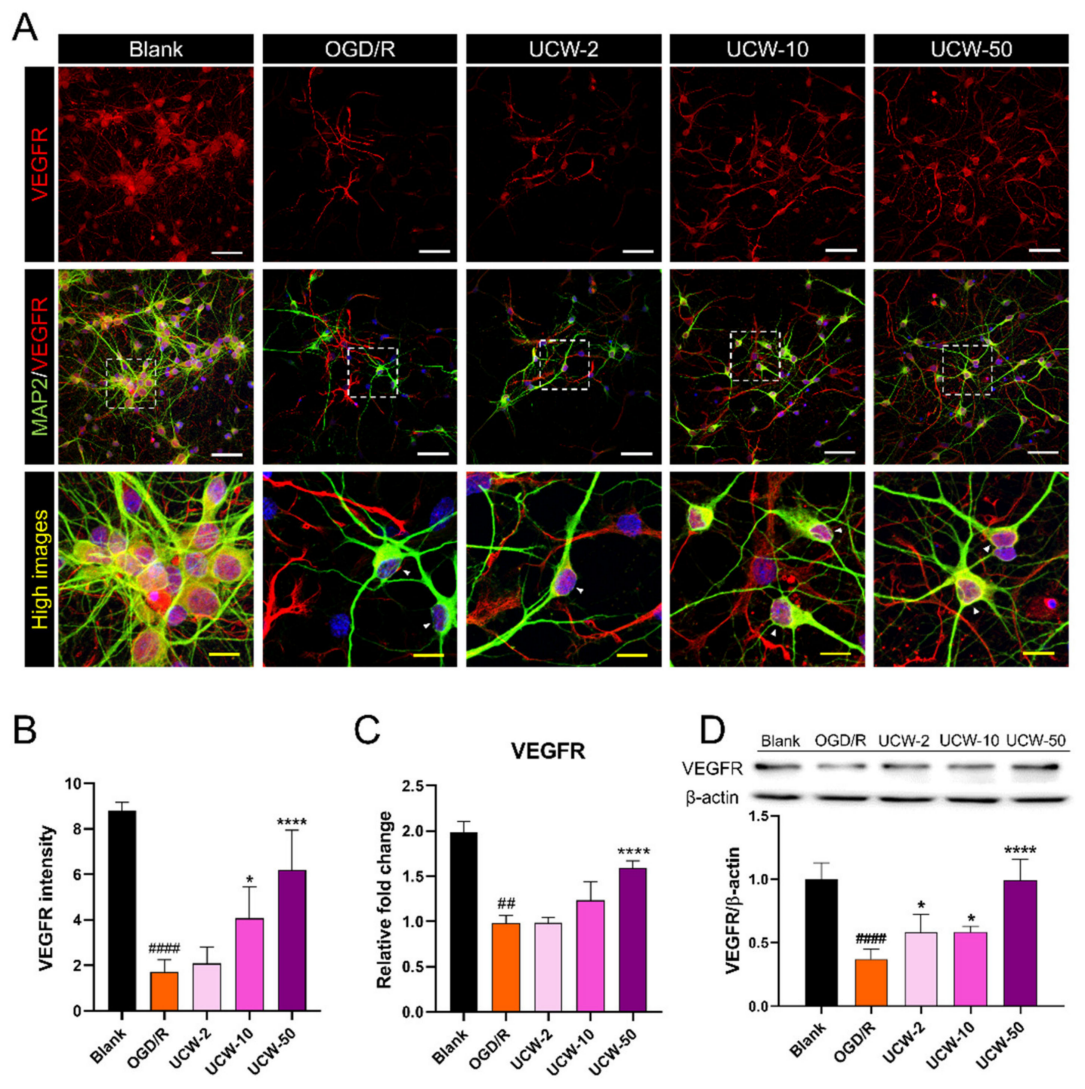
Angiogenic potential of UCW for OGD/R-induced hypoxic injury through enhancement of VEGF-VEGFR signaling. (**A**) Representative images of immunocytochemistry for MAP2 (green) and VEGFR (red) in blank, OGD/R injury, UCW + OGD/R injury. White scale bar = 50 µm, yellow scale bar = 10 µm. (**B**) Quantitative analysis of the relative VEGFR intensity in each group (*n* = 8). (**C**) The relative gene expression levels for *VEGFR* mRNA using real-time PCR in each group (*n* = 4). (**D**) Relative quantification of the VEGFR protein expression using Western blotting in each group (*n* = 4). Data are expressed as the mean ± SEM. Significant differences were analyzed via ordinary ANOVA with Tukey’s post-hoc analysis as follows: ^##^ *p* < 0.01 and ^####^ *p* < 0.0001 vs. blank group; * *p* < 0.05 and **** *p* < 0.0001 vs. the OGD/R group.

VEGF, which is considered a potent inducer of angiogenesis secreted by neurons, glial, and vascular endothelial cells, has not only neurotrophic activity but also enhances neuronal survival and neurite outgrowth and leads to a functional improvement in the pathogenesis of stroke, Alzheimer’s disease, spinal cord injury, and motor neuron diseases [19]. We first immunocytochemically evaluated the alterations in VEGF receptor (VEGFR) expression in neurons pretreated with UCW against OGD/R injury. Most MAP2^+^ cells were stained for VEGFR in the blank group; however, the amount of VEGFR-positive cells decreased after OGD/R exposure (Figure 3A). Meanwhile, quantitative analysis revealed that UCW pretreatment significantly enhanced VEGFR expression in a dose-dependent manner in OGD/R-injured neurons (Figure 3B). The mRNA level of *VEGFR* shows an increasing trend in UCW groups, but there was only a significant increase in the UCW-50 group compared to the OGD/R group (Figure 3C). Western blot analysis revealed a significant increase in VEGFR protein expression at all UCW doses compared to that in the OGD/R group (Figure 3D).

In addition, VEGF-stained neurons were quantified using immunocytochemistry. Confocal imaging showed that VEGF expression was similar to that of VEGFR (Figure S2A). There was a significant dose-dependent difference in the mean VEGF intensity between all UCW doses and the OGD/R group (Figure S2B). The mRNA level of *VEGF* was significantly increased in the two different UCW (10 and 50 µg/mL) compared to the OGD/R group (Figure S2C). These findings demonstrate that UCW improves angiogenesis via the VEGFR pathway by inducing neuronal VEGF secretion after OGD/R injury.

### 3.4. UCW Enhances MAP2 Signals Which Stained Neuronal Cell Bodies and Axons Exposed to OGD/R Injury in Primary Cortical Neurons

**Figure 4:**
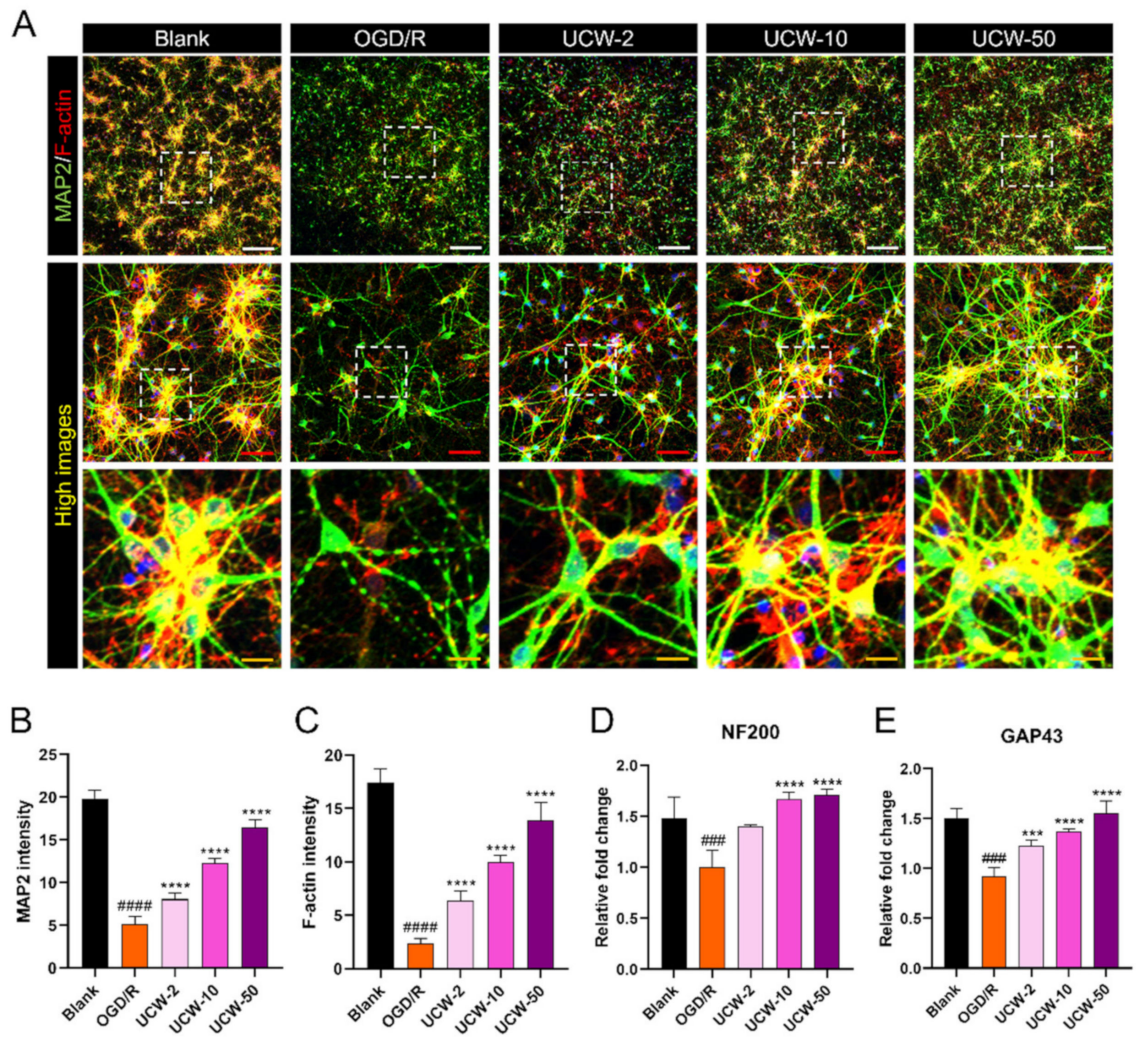
Effect of UCW on neuroprotection against OGD/R-induced injury in primary cortical neurons. (**A**) Representative images of immunocytochemistry for MAP2 (green) and F-actin (red) in blank, OGD/R injury, UCW + OGD/R injury. White scale bar = 100 µm, red scale bar = 50 µm, and yellow scale bar = 10 µm. (**B**,**C**) Quantitative analysis of (**B**) MAP2 and (**C**) F-actin intensity in each group (*n* = 8). (**D, E**) The relative mRNA expression level for (**D**) *NF200* and (**E**) *GAP43* using real-time PCR in each group (*n* = 4). Data are expressed as the mean ± SEM. Significant differences were analyzed via ordinary ANOVA with Tukey’s post-hoc analysis as follows: ^###^ *p* < 0.001 and ^####^ *p* < 0.0001 vs. blank group; *** *p* < 0.001 and **** *p* < 0.0001 vs. the OGD/R group.

Next, we studied the effect of UCW on neuroprotection in neurons exposed to OGD/R injury. OGD/R-induced axonal degeneration and fragmentation were clearly observed as breaks (dotted lines) in MAP2-stained mature neurons. Additionally, the neuronal population was markedly decreased, whereas the presence of UCW provided protection from OGD/R-induced decline in the cell population (Figure 4A). Dose-dependent effects of UCW were compared by quantifying the MAP2 intensity between groups, where a significant reduction was observed in the MAP2 intensity in the OGD/R groups. UCW pretreatment induced an increase in MAP2 intensity in a dose-dependent manner (Figure 4B). F-actin content in the leading edge of axons enhances neurite growth. Thus, with increasing doses of UCW, F-actin expression tended to increase and was more concentrated at the growth cones along which the neurites grew (Figure 4C). Furthermore, we confirmed the effect of UCW on the expression of regeneration-associated genes, including neurofilament 200-kDa (*NF200*) and growth-associated protein (*GAP43*). UCW induced a dose-dependent increase in *NF200* expression, with a significant difference between 10 and 50 µg/mL UCW doses and the OGD/R group (Figure 4D). The expression level of *GAP43* was also increased by UCW pretreatment, with significant and dose-dependent differences between all UCW doses and the OGD/R group (Figure 4E).

### 3.5. UCW Increases IGF-1R Expression on OGD/R-Induced Hypoxia Damage in Primary Cortical Neuron

**Figure 5:**
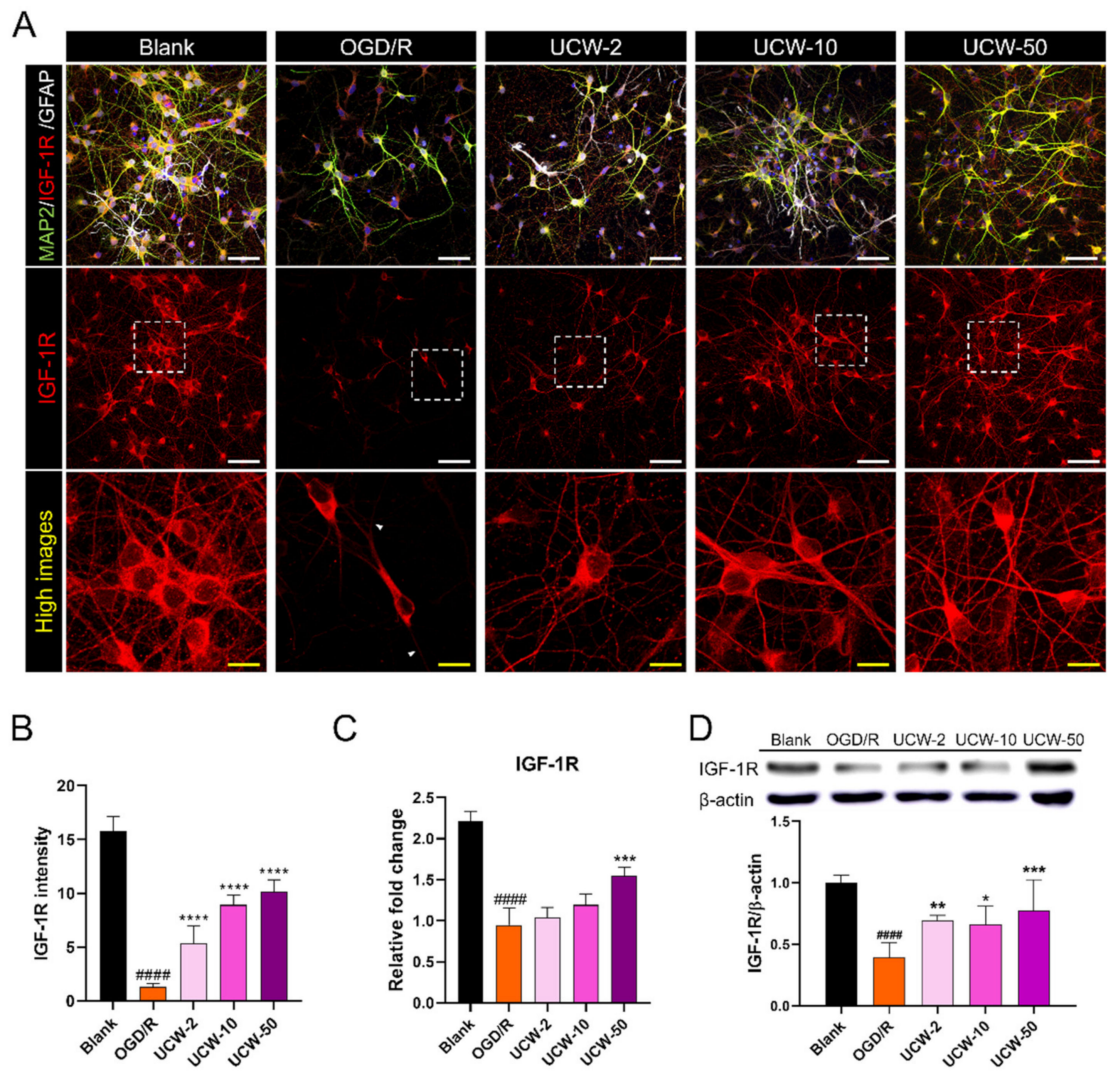
Neuroprotective effect of UCW on OGD/R-induced hypoxia damage by activating the IGF-1R signaling in primary cortical neurons. (**A**) Representative images of immunocytochemistry for IGF-1R (red), MAP2 (green), and GFAP (white) in blank, OGD/R injury, UCW + OGD/R injury. White scale bar = 100 µm, yellow scale bar = 10 µm. (**B**) Quantitative analysis of IGF-1R intensity estimated based on measurements obtained from 10 non-overlapping fields in each group (*n* = 10). (**C**) The relative mRNA expression level for *IGF-1R* using real-time PCR in each group (*n* = 4). (**D**) Relative quantification for the IGF-1R protein expression using Western blotting in each group (*n* = 4). Data are expressed as the mean ± SEM. Significant differences were analyzed via ordinary ANOVA with Tukey’s post-hoc analysis as follows: ^####^ *p* < 0.0001 vs. blank group; * *p* < 0.05, ** *p* < 0.01, *** *p* < 0.001, and **** *p* < 0.0001 vs. the OGD/R group.

IGF-1 and its receptor IGF-1R are produced in almost all cells and are essential for cell growth, survival, differentiation, and transformation [20]. Accumulating evidence for the beneficial role of IGF-1 in adult central nervous system (CNS) neurons has revealed its involvement in neuronal development, growth, and survival, including growth cone assembly and axonal formation [21]. Furthermore, IGF-1 stimulates angiogenesis through VEGF activation and has been shown to be involved in vascular remodeling in the brain [22,23]. Therefore, to examine the effects of UCW on IGF-1R expression after OGD/R injury, we evaluated the changes in IGF-1R expression at the gene and protein levels using immunocytochemistry, real-time PCR, and Western blotting. Immunocytochemical analysis revealed a reduced expression of IGF-1R in axons after OGD/R injury; however, there was no difference in the cell body of surviving neurons compared to that of the blank group. Meanwhile, UCW pretreatment induced an increase in IGF-1R expression in a dose-dependent manner (Figure 5A), and the mean IGF-1R intensity was significantly higher than that observed in the OGD/R group (Figure 5B). The mRNA expression levels of *IGF-1R* also showed a dose-dependent increase in UCW-pretreated neurons, but only UCW-50 showed a significant increase compared to the OGD/R group (Figure 5C). In addition, we quantified the protein expression of IGF-1R using Western blotting. The amount of IGF-1R was significantly higher (2-fold) in 50 µg/mL UCW-pretreated neurons post-OGD/R-induced hypoxia damage compared to that in the OGD/R group (Figure 5D). These results demonstrate that UCW pretreatment led to an increase in IGF-1R expression in OGD/R-induced hypoxia injured neurons.

### 3.6. UCW Prevents Synaptic Loss after OGD/R Injury in Primary Cortical Neurons

**Figure 6:**
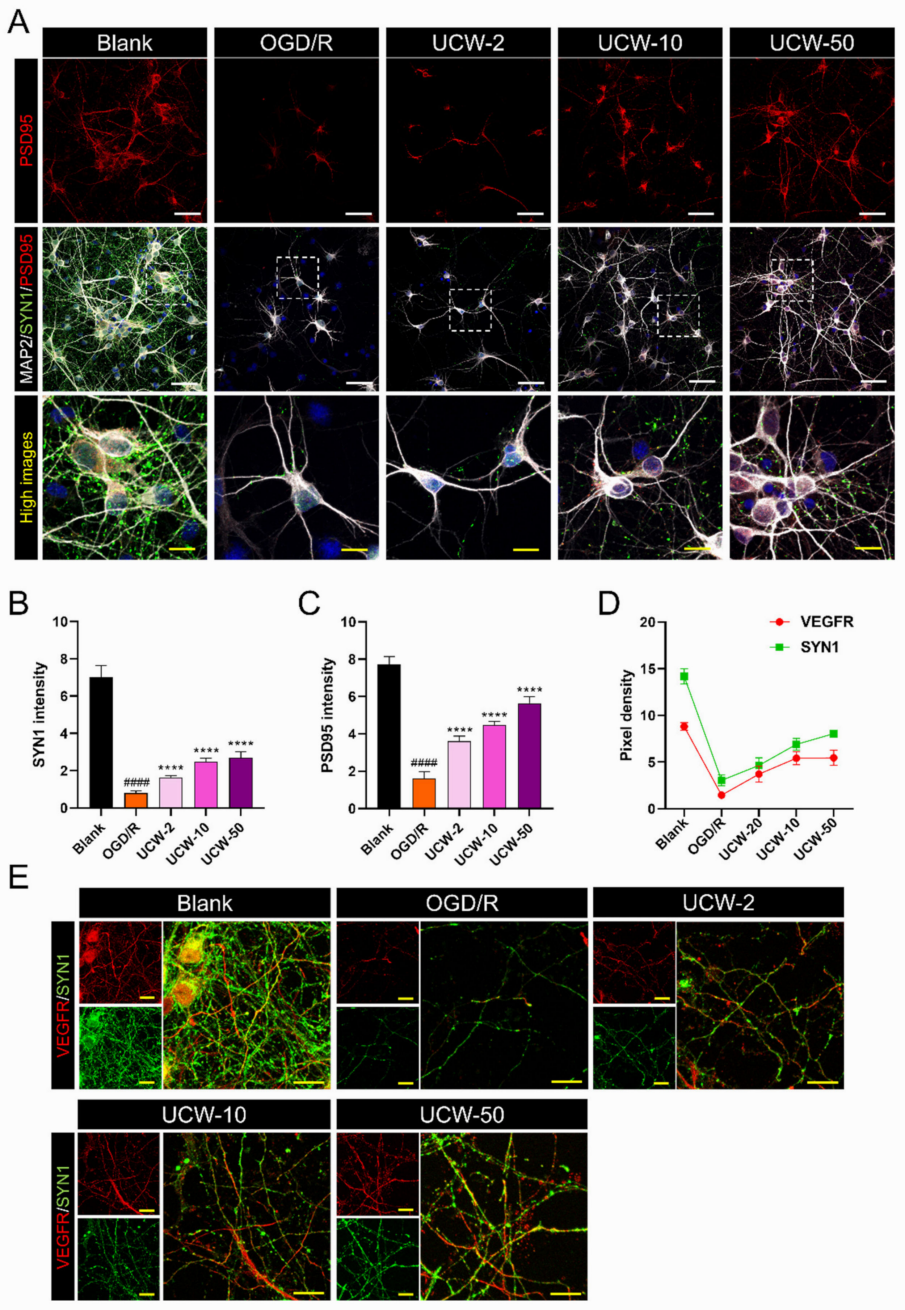
Preventing synaptic loss of UCW on OGD/R-injured neurons by upregulating synaptic proteins. (**A**) Representative images of immunocytochemistry for MAP2 (white), SYN1 (green), and PSD95 (red) in blank, OGD/R injury, UCW + OGD/R injury. White scale bar = 100 µm, yellow scale bar = 10 µm. (**B**,**C**) Quantitative analysis of (**B**) SYN1 and (**C**) PSD95 intensities in each group (*n* = 10). (**D**) Quantitative analysis of VEGF^+^ or SYN1^+^ pixel density in each group (*n* = 10). (**E**) Representative images of immunocytochemistry for VEGFR (red) and SYN1 (green) in each group. Yellow scale bar = 10 µm. Data are expressed as the mean ± SEM. Significant differences were analyzed via ordinary ANOVA with Tukey’s post-hoc analysis as follows: ^####^ *p* < 0.0001 vs. blank group; **** *p* < 0.0001 vs. the OGD/R group.

Synapses are critical neuronal junctions that transmit signals from one neuron to another utilizing synapsin1 (SYN1) and postsynaptic density protein (PSD95), which are synaptic proteins that are expressed throughout development and maturation [24]. In this study, we investigated synapse contact using triple-label fluorescence immunocytochemistry and confocal microscopy. SYN1 was abundantly expressed in the blank group but was barely expressed in OGD/R-injured neurons. However, UCW pretreatment induced an increase in SYN1 expression with the acceleration of neurite growth after OGD/R exposure. PSD95 expression followed a trend similar to that of SYN1 expression (Figure 6A). To quantitatively compare our observations, the relative intensities of SYN1 and PSD95 between groups were calculated. SYN1 and PSD95 intensities significantly decreased in the OGD/R group and increased in a dose-dependent manner after UCW pretreatment (Figure 6B,C). Furthermore, VEGF could be stored in the synaptic vesicle in neurons [25]. We used immunofluorescent staining of VEGFR and SYN1 to determine whether VEGFR expression differed within the synaptic vesicle of OGD/R-injured neurons. VEGFR was highly co-expressed with SYN1 but downregulated after OGD/R injury. Meanwhile, the pixel density of VEGFR appeared to be positively correlated with SYN1 and showed similar results with VEGFR and SYN1 expression depending on the gradual dose increment of UCW (Figure 6D,E).

## 4. Discussion

More than 17 million people worldwide succumb to vascular diseases each year, making it the leading cause of death [26]. Risk factors for stroke vary with age, including high blood pressure, smoking, diabetes, high cholesterol levels, excessive drinking, high-salt and high-fat diets, and lack of exercise [27]. Among them, hypertension is the most potent risk factor for stroke and a cause of life-threatening conditions [28]. Therefore, to manage and prevent high blood pressure, individuals seek alternative forms of hypertension care. Some herbs and supplements show promise as potential treatments for high blood pressure, but there is insufficient experimental evidence to support this claim.

UCW is a representative standardized herbal drug widely used for controlling blood pressure and heart stimulation. Previous studies on the effect of UCW on stroke have indicated that UCW has a transcription-activating effect on the NOS gene and a suppressing effect on the VCAM-1 gene in human endothelial cells [13]. Although VCAM-1 may be considered pharmacologically associated with stroke, it is closely involved in the progression of inflammatory and autoimmune disorders, including atherosclerosis, rheumatoid arthritis, multiple sclerosis, organ allografts, asthma, transplant rejection, and cancer [29,30]. In addition, the direct effects in a stroke model are yet to be confirmed.

Here, we demonstrated the effects of UCW in closely mimicking ischemic stroke models induced by oxygen glucose deprivation/re-oxygenation. We further used mature cortical neurons to study the molecular and cellular mechanisms underlying the changes in nerve cells of the brain tissue induced by UCW pretreatment and OGD/R injury. First, we found that treatment concentrations up to 50 µg/mL of UCW did not lead to neurotoxicity in primary cortical neurons, and the optimal doses of UCW were determined to be 2–50 µg/mL. UCW has great potential as a natural antioxidant that acts by regulating iNOS expression to alleviate the OGD/R-induced ROS levels in cortical neurons. Oxidative stress is one of the main mechanisms involved in the development of hypertension-induced vascular diseases [31].

In this study, we proposed a VEGF-dependent mechanism by which UCW proves to be beneficial in the prevention of hypoxic-ischemic neuronal injury that may occur after stroke. Although VEGFR expression remained similar to that in the blank group after OGD/R injury in neuron-supporting cells such as glial cells [32], VEGF-VEGFR signals were dramatically decreased in OGD/R-injured neurons. However, UCW has the potential to enhance angiogenesis and neurogenesis by upregulating VEGF-VEGFR expression. We also observed a robust increase in both axon extension and F-actin expression in UCW pretreated cortical neurons with OGD/R injury. Previous studies reported that IGF-1R is also crucial for angiogenesis, neurogenesis, and neuroprotection [33]. In particular, regarding its neurorestorative effect after stroke, IGF-1R may contribute to improved functional recovery and increased neurogenesis after treatment of stroke in rats with human marrow stromal cells [34]. Other studies have reported that IGF-1 reduced brain infarct volume and protected neurons via IGF-1R in the rat brain with ischemic stroke [35]. Thus, the activation of IGF-1R is reported to act as an ischemia-related determinant factor, including neurogenesis, synaptogenesis, and neurotransmission [36].

We also found that IGF-1R expression, analyzed by confocal imaging, was specifically abrogated in axons after OGD/R injury, whereas UCW dose-dependently promoted IGF-1R expression in the cell body and axons. Therefore, UCW is closely associated with IGF-1R and is a promising therapeutic medium against hypoxic-ischemic neuronal injury.

As a result, we demonstrated the effect of UCW on the prevention of synaptic loss after OGD/R injury, as it is a necessary process for functional improvement in the CNS. UCW treatment induced SYN1 and PSD95 expression, suggesting that UCW may reverse synaptic loss through the activation of these proteins.

Although UCW has been used since ancient times [12], there is insufficient scientific evidence that it can be utilized for the prevention of ischemia. In addition, UCW can cause unpleasant side effects such as drowsiness, heavy-headedness, and indigestion for clinically prolonged use [37]. Our data also cannot scientifically suggest the amount and time range of UCW used as their therapeutic target window. Furthermore, we only demonstrated the effect of UCW on the prevention of hypoxic damage in an in vitro model of hypoxic-ischemic-like injury with a short observation period using one cell type. Thus, further studies are needed to clarify the effect of UCW and to support the hypothesis of functional recovery in ischemic rodent models. Furthermore, the potential role of IGF-1R and VEGFR with respect to ischemia should also be evaluated in future studies to confirm its contribution to functional improvement after UCW administration. Nevertheless, our study was the first one to suggest the effects of UCW on its potential targets-IGF-1R and VEGF1R, contributing to neuroprotection and thereby preventing the hypoxic-ischemic injury that may be caused by stroke.

## 5. Conclusions

UCW prevents hypoxic-ischemic neuronal injury and synaptic loss due to elevated VEGF-VEGFR and IGF-1R likely in OGD/R-injured cortical neurons.

## Figures and Tables

**Table 1:** Primer sequences used for real-time PCR analysis.

Gene	5′-3′	Primer Sequence	NCBI Accession Numbers
*iNOS*	Forward	ATGGCTTGCCCCTGGAAGTT	NM_012611
	Reverse	TGTTGGGCTGGGAATAGCAC	
*VEGFR*	Forward	AGAGACCCGCGTTTTCAGAG	NM_013062
	Reverse	TGAGGTAGGCAGGGAGAGTC	
*NF200*	Forward	AACACCACTTAGATGGCGGG	NM_012607
	Reverse	ACGTGGAGCGTTCAGCAATA	
*GAP43*	Forward	TGCCCTTTCTCAGATCCACT	NM_017195
	Reverse	TTGCCACACAGAGAGAGAGG	
*IGF-1R*	Forward	GGGCCTGGTAATCATGCTGT	NM_052807
	Reverse	CATCCCGAAGGACCCTTGTC	
*VEGF*	Forward	GTACCTCCACCATGCCAAGT	NM_031836
	Reverse	AATAGCTGCGCTGGTAGACG	
*GAPDH*	Forward	CCCCCAATGTATCCGTTGTG	NM_017008
	Reverse	TAGCCCAGGATGCCCTTTAGT	

*iNOS*: inducible NOS; *VEGFR*; kinase insert domain receptor (KDR), also known as vascular endothelial growth factor receptor; *NF200*: neurofilament 200 kDa; *GAP43*, growth-associated protein 43; *IGF-1R*, insulin-like growth factor 1 receptor; *VEGF*: vascular endothelial growth factor; *GAPDH*, glyceraldehyde 3-phosphate dehydrogenase.

## Data Availability

Data is contained within the article and Supplementary Material.

## Supplementary Materials

The following are available online at: https://www.mdpi.com/article/10.3390/antiox11071388/s1. Video S1: Protocol for culturing rat cortical neurons. Video S2: Live-cell imaging of neurons over 24 h for PI-positive cells in red. Figure S1: Live and dead assay for UCW-only toxicity with increasing concentration. Figure S2: Immunocytochemical analysis of VEGF expression in UCW-pretreated and OGD/R-injured neurons.
