# Supplementary material for: Uwhangchungsimwon Inhibits Oxygen Glucose Deprivation/Re-Oxygenation-Induced Cell Death through Neuronal VEGF and IGF-1 Receptor Signaling and Synaptic Remodeling in Cortical Neurons
Source: Antioxidants (Basel). 2022 Jul 18;11(7):1388. doi: 10.3390/antiox11071388 (PMC9311511; doi:10.3390/antiox11071388)
Supplement: Supplementary file 1 [file antioxidants-11-01388-s001.zip › Supplementary materials.pdf]

Supplementary Materials

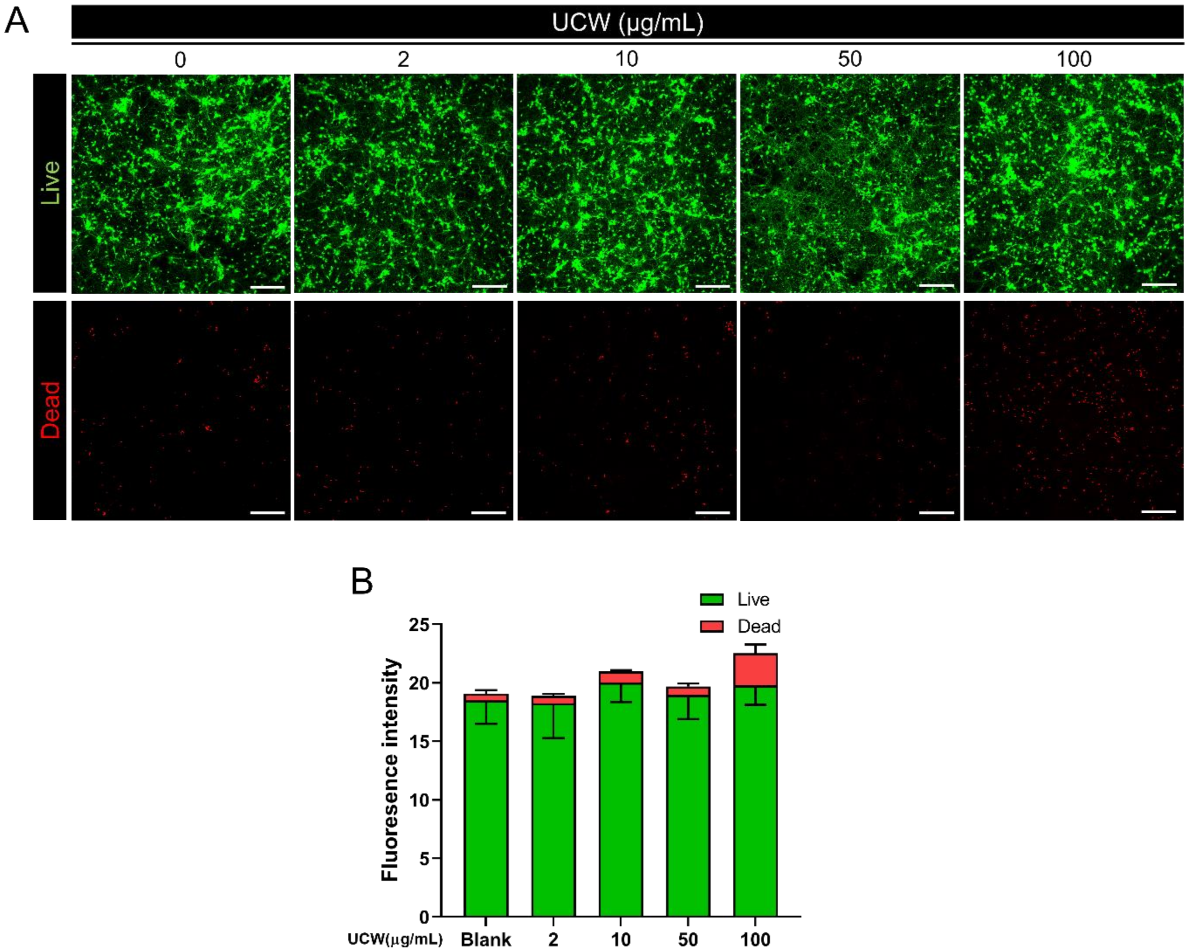

Figure S1. Live and dead assay for UCW-only toxicity with increasing concentration. (A) Representative images of live (green-stained) and dead (red-stained) cells in blank and different concentration of UCW (2, 10, 50 and 100 μg/mL). (B) Fluorescence intensity of live and dead cells in neurons pretreated with UCW for 48 h without OGD/R injury (n = 10). Data are expressed as the means ± SEM.

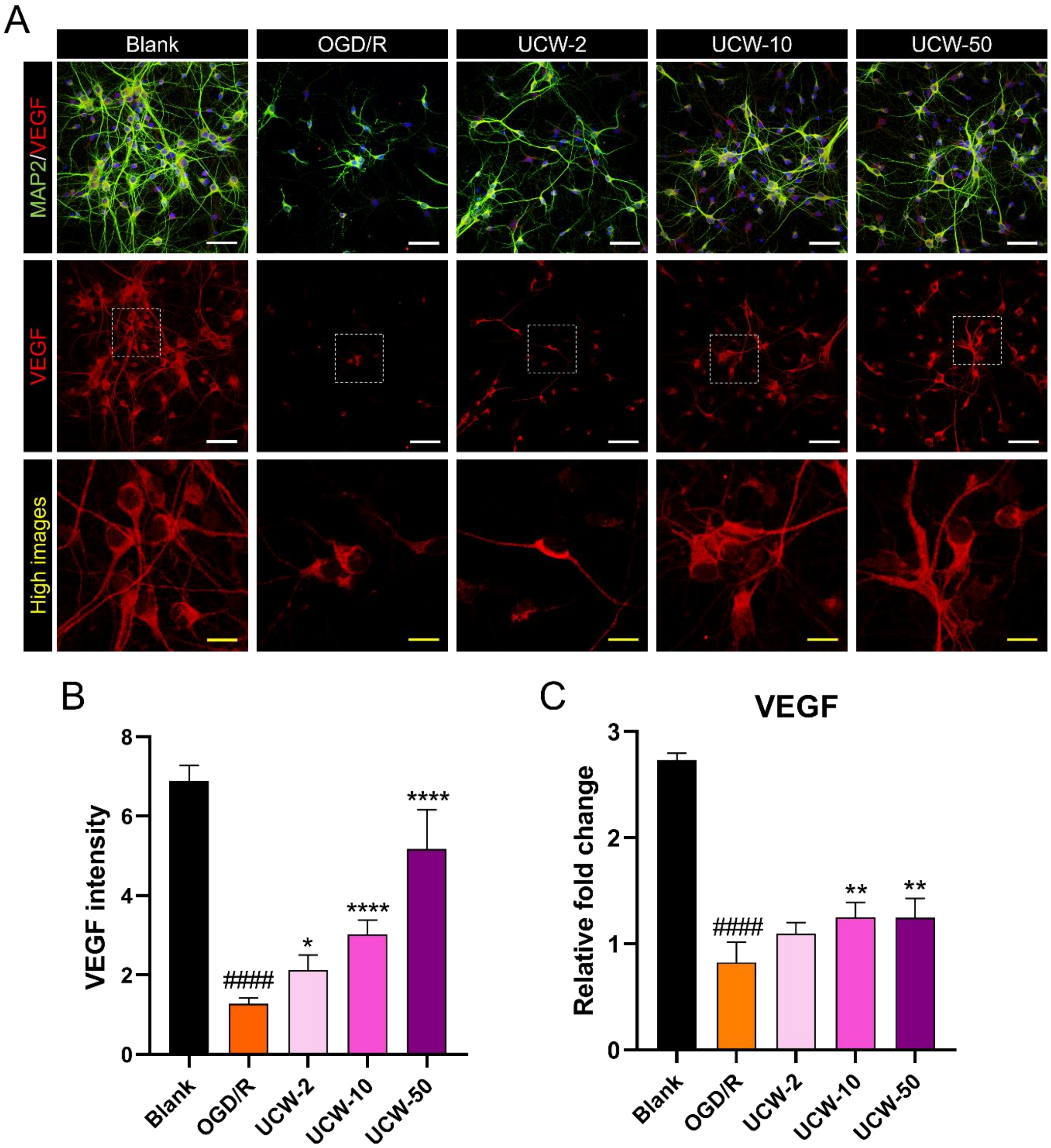

Figure S2. Immunocytochemical analysis of VEGF expression in UCW-pretreated and OGD/R-injured neurons. (A) Representative images of immunocytochemistry for MAP2 (green) and VEGF (red) in blank, OGD/R injury, UCW + OGD/R injury. (B) Quantitative analysis of the relative VEGF intensity in each group ($n = 10$). (C) The relative gene expression levels for *VEGF* mRNA using real-time PCR in each group ($n = 4$). Data are expressed as the means ± SEM. Significant differences indicated as $^{####}$ $p < 0.0001$ compared vs. the blank group; * $p < 0.05$, ** $p < 0.01$, and **** $p < 0.0001$ vs. the OGD/R group were analyzed via one-way ANOVA with Tukey's post hoc test.
